# Sources of Positivity in the Daily Life of Family Caregivers of Persons with Dementia

**DOI:** 10.1093/geroni/igab046.2468

**Published:** 2021-12-17

**Authors:** Kyungmi Lee, Breannlyn Archer, Kaitlyn Cox, Carolyn Pickering

**Affiliations:** 1 University of Alabama at Birmingham, Hoover, Alabama, United States; 2 The University of Alabama at Birmingham, Birmingham, Alabama, United States

## Abstract

Family caregivers often experience fatigue, burnout, and health complications yet also enjoy many aspects of caregiving that may benefit their well-being. This study identifies positive aspects of caregiving in the daily life experiences of dementia family caregivers in order to inform interventions to support caregivers’ well-being. This case study entails a secondary analysis of open-ended question data obtained from 165 family caregivers who answered daily diaries over 21 days (n = 2841 responses). We used content analysis to organize and elicit thematic categories from the data collected in response to the question “what was the best part of your day.” A final 762 responses were selected as meeting the “care” criteria for the study, with an inter-rater reliability of 91.6%. Data analysis revealed three major sources of daily positive aspects including: caregiver-focused, patient-oriented, and support-system based. The analysis also revealed seven different kinds of daily positive aspects, such as getting to enjoy time with the care recipient or getting to accomplish other non-caregiving tasks. Many of the positive aspects of caregiving reported were enabled by social support, but they were ultimately from how they utilized that support (e.g., getting alone time) that provided the positivity. The findings of this study demonstrate the important role social support plays in caregiving, as well as highlights other possible intervention targets to create easier, more positive days for family caregivers.

